# IDENTIFYING SENOLYTIC TARGETS OF DIETARY FLAVONOIDS IN RENAL CELLS BY THERMAL PROTEOME PROFILING

**DOI:** 10.1093/geroni/igad104.2997

**Published:** 2023-12-21

**Authors:** Quinn Strassheim, Reema Banarjee, Delaney Rutherford, Amit Dey, Dimitrios Tsitsipatis, Ruin Moaddel, Myriam Gorospe, Nathan Basisty

**Affiliations:** National Institute on Aging, National Institutes of Health, Baltimore, Maryland, United States; National Institute on Aging, National Institutes of Health, Baltimore, Maryland, United States; National Institute on Aging, National Institutes of Health, Baltimore, Maryland, United States; National Institute on Aging, National Institutes of Health, Baltimore, Maryland, United States; National Institute on Aging, National Institutes of Health, Baltimore, Maryland, United States; National Institute on Aging, National Institutes of Health, Baltimore, Maryland, United States; National Institute on Aging, National Institutes of Health, Baltimore, Maryland, United States; National Institute on Aging, National Institutes of Health, Baltimore, Maryland, United States

## Abstract

Senescent cell accumulation is a known driver of aging and age-related pathologies. Clearance of senescent cells is a promising approach to increase longevity and reduce multiple age-related diseases in humans. Flavonoid compounds are present in fruits and vegetables, of which certain flavonoids (e.g., quercetin and fisetin) have demonstrated senolytic, or ‘senescent-cell-killing’, activity in culture and in mice. However, the cellular mechanism of senolysis and the efficacy of these compounds are not known in renal epithelial cells. Here, we combine flavonoid drug screening, targeted metabolomics, and thermal proteome profiling (TPP) with analysis by mass spectrometry (MS) to explore protein targets of senolytic drugs in senescent human renal cortical epithelial cells and human renal proximal tubular epithelial cells. Senescence was induced by exposure to ionizing radiation and the senescence phenotype was validated through a rigorous panel of senescence and viability markers. Proliferating and senescent renal epithelial cells were screened with a panel of 8 flavonoids to identify senolytic drugs and their concentrations for selectively killing senescent cells. Targeted MS assays were developed to assess intracellular drug uptake, a potential mechanism of senescent-specific killing. To identify protein targets of the senolytic flavonoids in senescent cells, we performed a variation of TPP in combination with liquid chromatography (LC)-MS/MS proteomics analysis, carefully controlling for non-senolytic interactions by excluding proteins bound by a non-senolytic flavonoid. The results of this study pave the way for the development of a more specific generation of senolytic compounds and identify novel candidate senolytic pathways engaged by dietary flavonoids.

